# The putative type 4 secretion system effector BspD is involved in maintaining envelope integrity of the pathogen *Brucella*

**DOI:** 10.1128/msphere.00232-24

**Published:** 2024-10-10

**Authors:** Maren Ketterer, Petra Chiquet, Mara Esposito, Jaroslaw Sedzicki, Maxime Québatte, Christoph Dehio

**Affiliations:** 1 Biozentrum, University of Basel, Basel, Switzerland; University of Kentucky College of Medicine, Lexington, Kentucky, USA

**Keywords:** type 4 secretion system effector, BspD, *Brucella*, envelope integrity, outer membrane, stress tolerance, EipA, type 4 secretion system

## Abstract

Brucellosis is a debilitating disease caused by the Gram-negative, facultative intracellular zoonotic pathogen *Brucella.* En route to its intracellular replicative niche, *Brucella* encounters various stressful environments that may compromise envelope integrity. Here we show that the proposed type 4 secretion system (T4SS) effector BspD is a conserved protein of the Rhizobiales, which does not show signs of co-evolution with the presence of a T4SS or a certain lifestyle. We further present data indicating that BspD is critical for the envelope integrity of *Brucella abortus* in the stationary phase and in the presence of EDTA, a compound known to destabilize the outer membrane. Deletion of *bspD* resulted in abnormal bacterial morphologies, indicating its involvement in maintaining envelope integrity. Additionally, the absence of BspD led to the formation of fewer and smaller intracellular microcolonies in a macrophage infection model. From our observations, we propose that BspD of *B. abortus* is critical for preserving the integrity of the bacterial envelope, particularly under stressful conditions, which may enhance *Brucella*’s ability to survive within host cells.

**IMPORTANCE:**

Brucellosis, caused by the intracellular pathogen *Brucella*, poses a significant health threat. Understanding how *Brucella* adapts to stressful environments is crucial. This study unveils BspD, a conserved protein within the Rhizobiales order, as a key player in maintaining *Brucella*'s envelope integrity. Remarkably, BspD’s presence within the Rizobiales appears independent of the presence of a T4SS or a specific lifestyle. Deletion of *bspD* resulted in compromised envelope integrity, abnormal bacterial morphologies, and reduced intracellular microcolony formation. These findings underscore BspD’s critical role, particularly in stressful conditions like the stationary phase and EDTA exposure, and highlight its significance for the survival of *Brucella* within host cells. This elucidation deepens our understanding of *Brucella* pathogenesis and may inform future therapeutic strategies against brucellosis.

## INTRODUCTION

Rhizobiales, an order of diverse Gram-negative α-Proteobacteria, is specialized to a wide range of niches; spanning from free-living bacteria to plant symbionts and intracellular mammalian pathogens (1, 2). The genus *Brucella* comprises the facultative intracellular zoonotic pathogens *Brucella abortus*, *Brucella suis*, and *Brucella melitensis*, which are the causative agents of brucellosis, a severe zoonosis manifesting through a broad range of symptoms, encompassing undulant fever and flu-like episodes (3). As a facultative intracellular pathogen, *Brucella* has evolved strategies to survive and replicate within host cells. Following uptake, the *Brucella*-containing vacuole (BCV) is trafficking along the canonical endocytic route to at least the late endosomal stage (4–8). Acidification of the BCV serves as a signal for triggering expression of the bacterial type 4 secretion system (T4SS) VirB, which then facilitates rerouting of the BCV to the endoplasmic reticulum (ER), where bacteria establish their replicative niche (4, 7–11). T4SSs are pivotal virulence factors of numerous host-interacting bacteria; they represent large macromolecular complexes spanning the bacterial envelope that facilitate the export of cargo from the bacterial cytoplasm, such as the translocation of toxins to the extracellular environment or of bacterial effector proteins directly into host cells (reviewed in reference 12). Deletion of any gene encoding an essential component of the VirB machinery incapacitates the mutant bacteria to reach the ER and to replicate intracellularly, demonstrating the essential role of the T4SS in *Brucella*’s intracellular lifestyle (7, 13). In contrast, none of the growing list of putative T4SS effector proteins has been found to be individually essential for intracellular replication or survival (14). Despite the ample evidence for a central role of the VirB T4SS for *Brucella*’s virulence (15–17), we are still lacking a molecular understanding of how the machinery works, including the nature of translocation motifs and recruitment mechanisms of the effectors and the process of their translocation.

Before reaching the ER-derived replication niche, bacteria trafficking within the BCV encounter a challenging and ever-changing environment, including acidification, the action of cationic peptides and lytic enzymes, and the activity of reactive oxygen species (18, 19). A key component to withstand these chemical stressors is *Brucella*’s cell envelope, which consists of an inner membrane, an outer membrane, and the periplasmic space with the peptidoglycan layer in between. *Brucella* is long known for its remarkable resistance to chelators, detergents, and cationic peptides (20, 21). However, we are only now beginning to gain insights into the molecular mechanisms facilitating stress resistance and maintenance of envelope integrity. Some of the involved components are the periplasmic proteins EipA (22) and EipB (23), the TamB homolog MapB (24), and *Brucella*’s distinct lipopolysaccharide (LPS) (21, 25). They have been shown to confer resistance to several cell wall stressors, such as hyperosmotic and acidic environments, cell wall-active antibiotics, metal chelators, and cationic peptides, and are important for survival within the host (21–25).

Interestingly, the putative T4SS effector protein BspD (BAB1_1611) of unknown function (26, 27) is encoded on chromosome 1 directly downstream from the gene encoding the envelope integrity protein EipA (22). This locus architecture prompted us to test for a potential role of BspD in envelope integrity. Through a combination of *in silico* analysis, *in vitro*, and *in cellulo* experiments, we show that (i) BspD is a conserved protein confined to the Rhizobiales; (ii) the deletion of *bspD* confers a mild but significant growth defect in rich broth in the stationary phase; and (iii) the *bspD* mutant displays sensitivity to EDTA. We conclude that BspD has a conserved function in envelope homeostasis that is likely independent of any T4SS-related function. Further, we show that deletion of *bspD* compromises intracellular microcolony formation in macrophages, highlighting the role of BspD in mediating stress tolerance *in cellulo*, but this mutant phenotype could also reflect the proposed role of BspD as a T4SS effector.

## RESULTS

### The putative T4SS effector BspD is conserved in Rhizobiales

**Fig 1:**
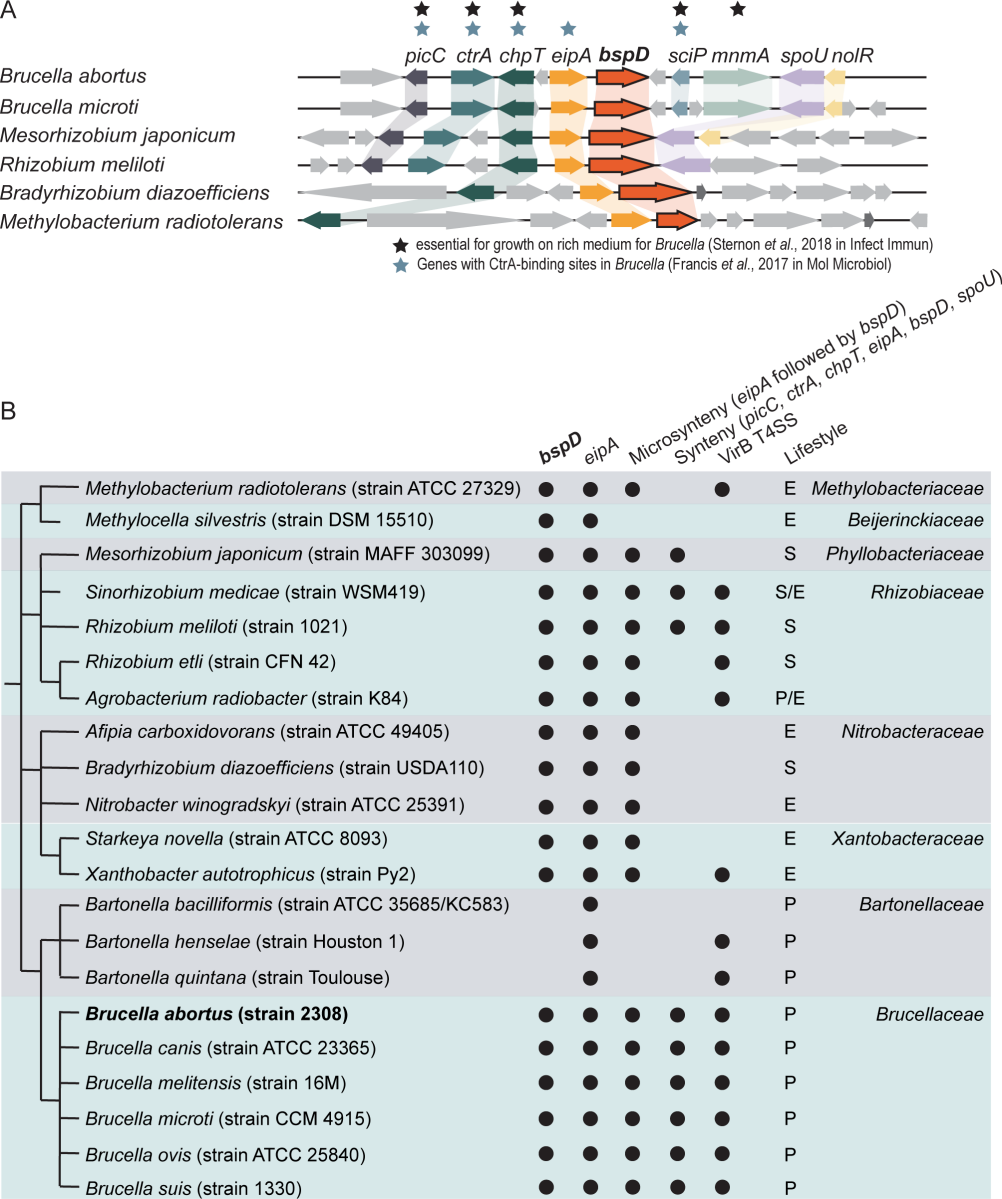
The putative effector BspD is conserved in Rhizobiales. (**A**) *bspD* is co-conserved with *picC*, *ctrA*, *chpT*, *eipA*, and *spoU* or only with *eipA* in the same locus in different subsets of Rhizobiales. Gene synteny redrawn from SyntTax with either *bspD* or *eipA* used as bait. Black stars depict genes reported to be essential for growth on rich medium for *Brucella*. Blue stars mark genes with reported CtrA-binding sites in *Brucella*. (**B**) Simplified phylogenetic tree of the genus Rhizobiales based on the species phylogeny of hierarchical orthologous groups (HOGs) as defined by the OMA browser. Presence of *bspD* (BAB1_1611) and *eipA* (BAB1_1612) orthologs based on their respective HOGs is indicated with a filled circle. Co-conservation with *eipA* alone in the same locus (microsynteny) or with *picC*, *ctrA*, *chpT*, *eipA*, and *spoU* in the same locus (synteny) is depicted with a filled circle deduced from SyntTax. Presence of VirB T4SSs is depicted with a filled circle as inferred from the SecReT4 database. The different lifestyles (S, P, and E) were inferred from literature. E, environmental; P, animal pathogen; S, plant associated.

In *Brucella*, the gene encoding the putative T4SS effector BspD is located directly downstream to the genes encoding the envelope integrity protein EipA and the essential cell cycle response regulator CtrA and its upstream regulator, the histidine phosphotransferase ChpT (22), as well as PicC, an essential protein of unknown function (14). Located downstream of *bspD* are genes coding for the essential transcription factor SciP (14, 28), MnmA, the RNA methyltransferase SpoU, and the transcriptional regulator NolR, which is essential for virulence (29, 30) (Fig. 1A). Several genes surrounding *bspD* have CtrA-binding sites (28) and are essential for growth of *Brucella* on rich medium (14) (Fig. 1A). Prompted by the conserved synteny (Fig. 1A), we wanted to explore the evolutionary history of BspD in more detail. Therefore, we analyzed the presence of the *bspD* gene and the conservation of the synteny with *picC*, *ctrA*, *chpT*, *eipA*, and *spoU*, as well as the microsynteny with *eipA* within the entire Alphaproteobacteria. Using the OMA browser (31, 32) to search for *bspD* orthologs, we found that *bspD* was confined to Rhizobiales and conserved in a majority of its member genera and species (Fig. 1B). Moreover, its presence does not correlate with the presence of a VirB T4SS or a particular lifestyle. For instance, BspD homologs are found in many soil-dwelling species such as *Methylocella silvestris* and *Strakeya novella*, as well as classical plant symbionts such as *Rhizobium meliloti* and *Mesorhizobium japonicum*, while the mammalian pathogens comprising *Bartonellae* do not encode BspD (Fig. 1B). Other than *bspD*, the genes coding for EipA, CtrA, and ChpT are not confined to Rhizobiales and are also found in the Sphingomonadales, Caulobacterales, Parvulaculales, and the Rhodobacterales (22). Within species of the Rhizobiales, the synteny of *bspD* with at least *picC*, *eipA*, *chpT*, and *ctrA* extends beyond the genus *Brucella* also to species of the *Phyllobacteriaceae* and *Rhizobiacea*, such as *Mesorhizobium japonicum* and *Rhizobium meliloti*, respectively (Fig. 1A and B), while in other members of Rhizobiales, the microsynteny is limited to *eipA* (Fig. 1A and B) (33). A protein sequence alignment further revealed that BspD of *Brucella* and *Ochrobactrum* is truncated compared to orthologs of other species (Fig. S1), which coincides with *Brucellaceae* forming a robust cluster in a protein-based phylogenetic analysis (Fig. S2).

### Deletion of *bspD* leads to growth defect in rich broth

Given the conserved linkage of *bspD* with genes encoding the envelope integrity protein EipA (22) or the essential growth regulators CtrA/ChpT (14, 28), we aimed to test whether BspD might have a conserved function in growth control or envelope homeostasis.

**Fig 2:**
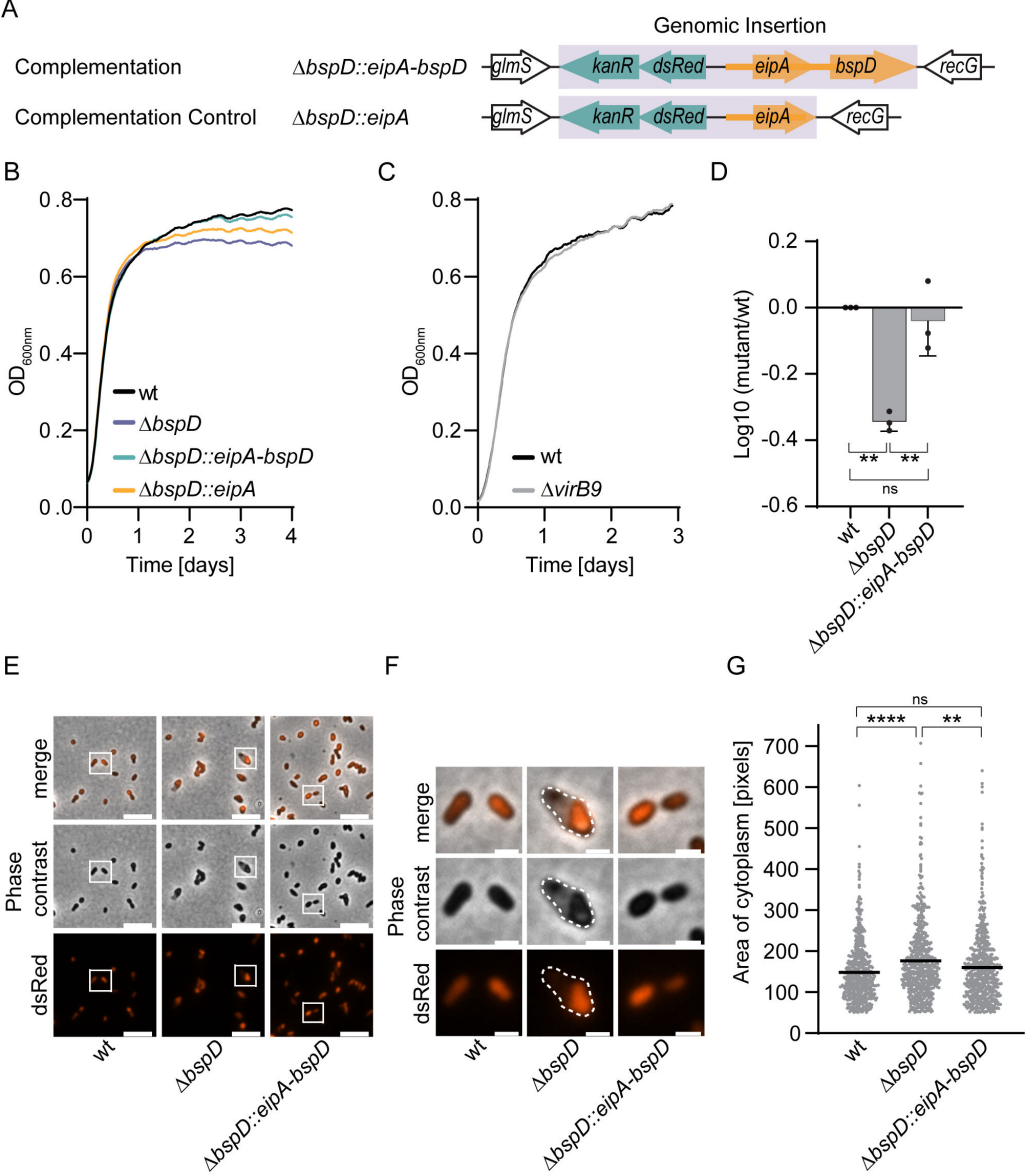
Deletion of *bspD* results in a stationary phase survival defect of *Brucella*. (**A**) Overview of Δ*bspD* complementation strategy. To ensure native expression levels the 5′ non-coding regions of *eipA* and *bspD* (orange thick line), encoding potential promoters were included. Violet shaded area denotes genomic insertion. Black outline denotes genomic context. Teal denotes *kanR* and *dsRed* genes. Orange denotes *eipA* and *bspD* with 5′ non-coding regions. (**B and C**) Optical density of indicated *Brucella* strains grown in 150-µL TSB in a 96-well plate over 96 h (**B**) or 72 h (**C**), as indicated, with a starting OD_600_ of 0.08. The OD_600_ was measured every 10 min in a synergy H1 plate reader. Lines depict the means of three independent experiments (**B**) or two independent experiments with technical triplicate (**C**) with a second-order smoothing of 15 neighbors applied in GraphPad Prism (version 9.3.1). (**D**) Comparison of recovered CFU of wild type, deletion mutant, and complemented mutant from panel **B** at 96 h. Cultures were serially diluted and plated on tryptic soy agar plates. CFUs were enumerated after 3–4 days of incubation at 37°C and the log10 survival was calculated by the deletion of mutant CFU per milliliter by wt CFU per milliliter. Corresponding CFU enumerations and survival ratios in percent are displayed in Fig. S3B and C, respectively. (**E**) Phase contrast and fluorescence micrographs of wt, deletion mutant, and rescue cultured in TSB for 96 h before fixation. Insets in Δ*bspD* highlight bacterial cells with plasmolysis. Scale bar = 5 µm. Representative images of three independent replicates. (**F**) Enlarged insets of panel** D** showing bacteria with apparent plasmolysis. Broken line denotes the outline of the bacterium. Scale bar = 1 µm. (**G**) Evaluation of bacterial morphology of bacteria recovered from panel** B** at 96 h. The area of the dsRed signal of 200 single bacteria each from three independent experiments was measured using CellProfiler as a proxy for bacterial size. The line marks the median area of the cytoplasm. *dsRed*, *dsRed* under *P_aphT_*.; *kanR*, kanamycin resistance cassette under *P_aphT_.*; ns, not significant; TSB, tryptic soy broth; wt, wild type.

To accomplish this, we generated a Δ*bspD* deletion mutant and employed a previously described miniTn7-based strategy for integration of a single chromosomal copy of a *bspD* rescue construct between *glmS* and *recG* of chromosome 1 (26) (Fig. 2A). Considering the possibility that *eipA* and *bspD* might constitute a bi-cistronic operon co-transcribed from the upstream located CtrA-controlled *eipA* promoter (22), we integrated a fragment encoding both genes, including the *eipA* promoter (Δ*bspD::eipA-bspD*), to ensure native expression of BspD. To eliminate the potential influence of an additional copy of *eipA*, we also constructed an isogenic Δ*bspD* mutant strain containing an insertion solely for *eipA*, along with its promoter region (Δ*bspD::eipA*) (Fig. 2A).

We then tested the growth of the various isogenic *B. abortus* strains in tryptic soy broth (TSB) over 96 h in comparison to the parental wild-type strain. Compared to the wild-type strain and the complemented mutant strain Δ*bspD::eipA-bspD*, the Δ*bspD* mutant and the Δ*bspD::eipA* control strain displayed a growth defect in rich broth. This defect was characterized by an earlier entry into the stationary phase, indicated by an earlier inflection point and a lower maximum OD_600_ (Fig. 2B; Table 1; Fig. S2A).

**TABLE 1:** Growth dynamics extracted from the Gompertz fitting displayed in Fig. S2A to the data presented in Fig. 2B

	wt	Δ*bspD*	Δ*bspD*::*eipA-bspD*	Δ*bspD*:*eipA*
*R* squared (goodness of fit)	0.9531	0.9659	0.9489	0.9661
Inflection point (h)	6.9576 (±0.2856)	4.7832 (±0.1584)	6.6672 (±0.2808)	5.0064 (±0.1704)
Maximum population (OD_600_)	0.7462 (±0.0031)	0.6857 (±0.0021)	0.7392 (±0.0031)	0.7122 (±0.0022)

Notably, a Δ*virB9* mutant displayed comparable growth to wild-type bacteria (Fig. 2C), suggesting that the observed growth defect of the Δ*bspD* mutant is independent of a functional T4SS in broth. CFU counts performed at the end of the experiment showed that mutant levels were approximately 50% lower than those of wild type (Fig. 2D; Fig. S3B and C). Further, mutant bacteria displayed altered morphologies, such as elongation, swelling, and plasmolysis (contraction of the cytoplasm visualized by cytoplasmic dsRed), compared to wild-type bacteria, which is reflected by a significant increase in the area covered by the cytoplasm as a proxy for bacterial size (Fig. 2E through G; Fig. S4A).

### The Δ*bspD* mutant is destabilized by EDTA

**Fig 3:**
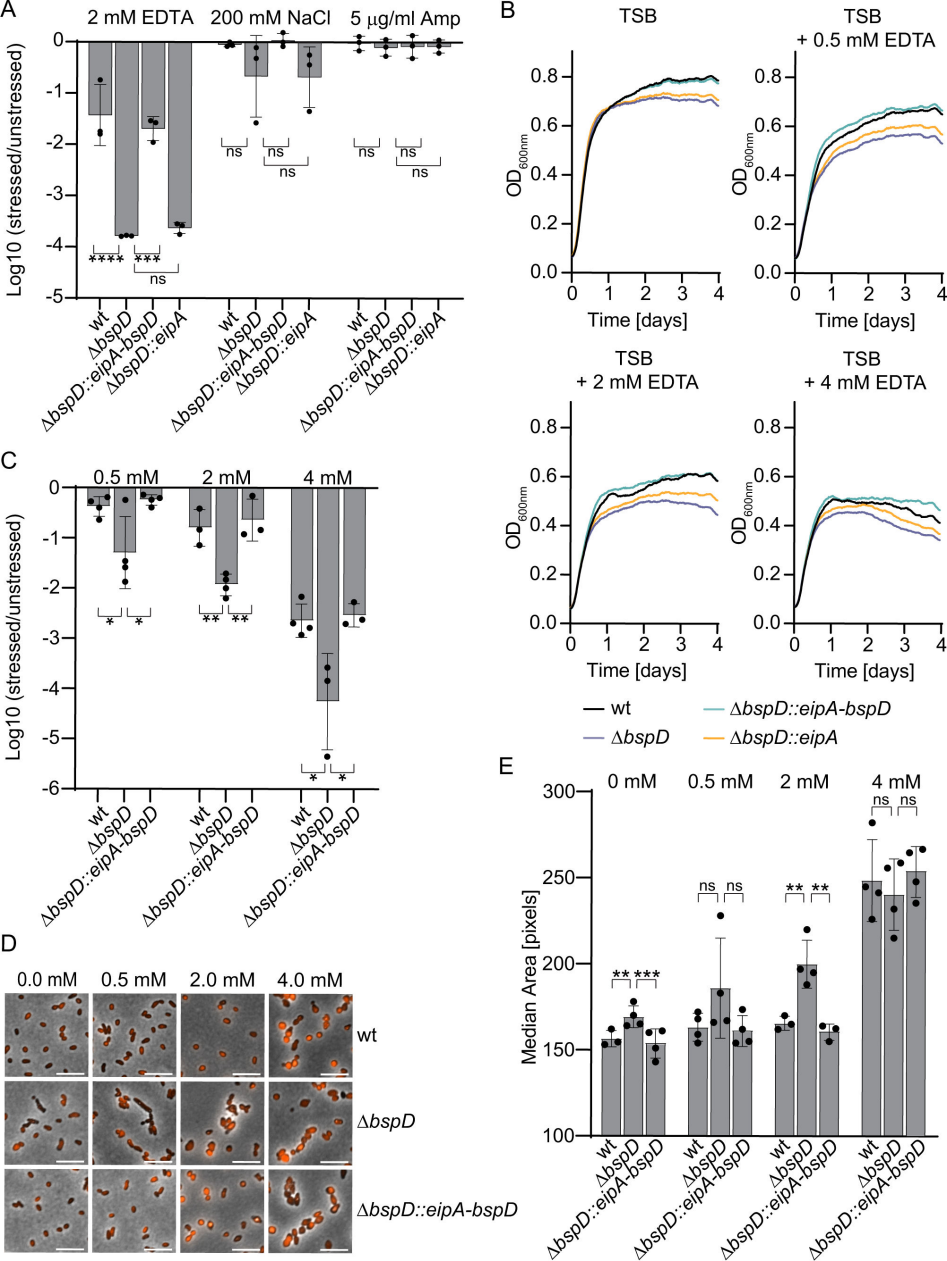
Deletion of *bspD* leads to a survival defect in presence of EDTA. (**A**) Plate stress assay to assess survival in the presence of envelope stressors. Indicated *Brucella* strains were grown to stationary phase, and the density was adjusted to 1 × 10^8^ CFU/mL. Samples were serially diluted and spotted onto plain TSA plates or TSA plates supplemented with either 2 mM EDTA, 200 mM NaCl, or 5-µg/mL ampicillin. CFUs were enumerated after at least 3 days of growth at 37°C. The *Y*-axis shows for each strain the log10 reduction for the ratio of CFUs on supplemented TSA vs CFUs on plain TSA. Dots represent means of two technical replicates of three independent experiments. Corresponding CFU enumerations are displayed in Fig. S3D. (**B**) Optical density of indicated *Brucella* strains grown in 150 µL plain TSB or TSB supplemented with either 0.5 , 2.0 , or 4.0 mM EDTA in a 96-well plate over 96 h with a starting OD_600_ of 0.08. The OD_600_ was measured every 10 min in a synergy H1 plate reader. Depicted is one representative experiment of four independent replicates. Data are displayed with a second-order smoothing of 15 neighbors applied in GraphPad Prism (version 9.3.1). (**C**) Relative survival of mutants vs wt bacteria recovered after 96-h exposure to different EDTA concentrations corresponding to panel** B**. Samples were serially diluted and plated on TSA plates. After 3–4 days of incubation at 37°C, CFUs were enumerated and the log10 survival was calculated by deletion of mutant CFU per milliliter by wt CFU per milliliter. Outliers were removed as described in Materials and Methods. Corresponding CFU enumerations are displayed in Fig. S3E. *n* = 4. (**D**) Representative micrographs of bacteria recovered from panel **B**. Red denotes dsRed; black denotes phase contrast. Scale bar = 5 µm. (**E**) Evaluation of bacterial morphology exposed to different EDTA concentrations for 96 h. The area of the dsRed signal of >500 single bacteria from four independent experiments was measured using CellProfiler as a proxy for bacterial size. Displayed are the median areas measured in the independent experiments. Outliers were removed as described in Materials and Methods. TSA, tryptic soy agar.

Next, we investigated if the Δ*bspD* mutant might be compromised in envelope homeostasis using the same conditions as previously described for the Δ*eipA* mutant (22). To this end, we tested the sensitivity of the Δ*bspD* mutant vs wild type to metal chelation (EDTA), hyperosmotic stress (high NaCl concentration), and peptidoglycan stress (ampicillin). The Δ*bspD* mutant displayed significant sensitivity to EDTA in the stress plate assay when compared to growth in the absence of any stressor, but only mild sensitivity to high NaCl concentrations (Fig. 3A; Fig. S3D). There was no difference in the sensitivity of the Δ*bspD* mutant to ampicillin (Fig. 3A; Fig. S3D).

To further characterize the nature of the increased sensitivity of the Δ*bspD* mutant to EDTA, we grew bacteria in rich broth in the presence of increasing EDTA concentrations for 4 days, continuously measuring the OD_600_, and evaluating the CFU per milliliter and morphology at endpoint (Fig. 3B through E). We observed that the stationary phase defect of the Δ*bspD* mutant in TSB was exacerbated in the presence of low EDTA concentrations (Fig. 3B), which correlated with a survival defect based on CFU counting at the endpoint (Fig. 3C; Fig. S3E). We further observed that the bacterial shape and size of wild-type bacteria were stable from 0 to 2 mM EDTA, while the size of Δ*bspD* mutant bacteria increased steadily and showed aberrant morphologies with increasing EDTA concentrations, such as cell elongation, swelling, and rounding (Fig. 3D and E), indicating a destabilization of the bacterial envelope. These findings suggest that BspD is required for envelope integrity in the stationary phase and under outer membrane stress conditions. Notably, the outcomes we observed in the presence of hyperosmotic stress or ampicillin do not indicate a broad destabilization in the Δ*bspD* mutant. Instead, they imply a more specific role of BspD in envelope integrity.

### The Δ*bspD* mutant is compromised in the formation and growth of intracellular microcolonies

**Fig 4:**
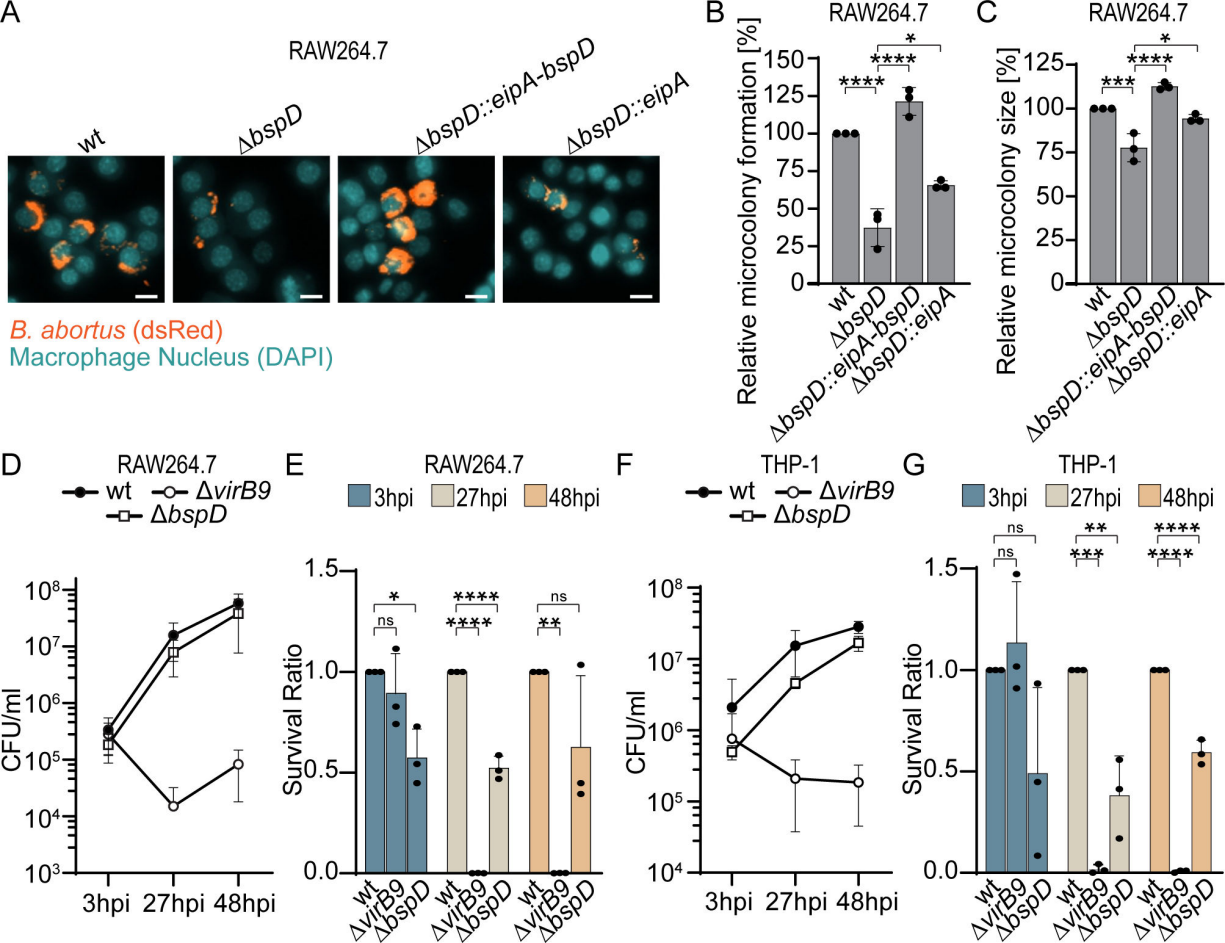
The Δ*bspD* mutant is compromised in intracellular survival and replication within macrophages. (**A**) Representative images of infected RAW264.7 macrophages. RAW264.7 macrophages were infected with an MOI of 50 for 27 h. After fixation and staining of host cell nuclei with 4′,6-diamidino-2-phenylindol (DAPI), samples were imaged using a Molecular Devices ImageXpress microscope. Cyan denotes host cell nuclei; orange denotes dsRed-expressing *Brucella*. Scale bar = 10 µm. *n* = 3. (**B**) Relative microcolony formation of different *Brucella* strains normalized to wt. Relative microcolony formation corresponds to the quantity of macrophages with identified microcolonies. (**C**) Relative microcolony size formed by different *Brucella* strains expressed as percentage relative to wild-type level. The relative microcolony size of the mutants based on the median size of about 7,000 identified infection sites in each experiment was calculated in relation to the wt. (**D**) Enumeration of bacteria from infected RAW264.7 macrophages at 3, 27, and 48 hpi. Macrophages were infected with an MOI of 50 as described. At the indicated time points, the macrophages were lysed, and bacteria were recovered and serially diluted on agar plates for enumeration of CFU per milliliter. *n* = 3. (**E**) Data presented in panel** D** displayed as survival ratio at the given time point normalized to the wild-type CFU. (**F**) Enumeration of bacteria from infected THP-1 macrophages at 3, 27, and 48 hpi. Macrophages were infected with an MOI of 50 as described. At the indicated time points, the macrophages were lysed, and bacteria were recovered and serially diluted on agar plates for enumeration of CFU per milliliter. *n* = 3. (**G**) Data presented in panel F displayed as survival ratio at the given time point normalized to the wild-type CFU. All data are displayed as mean, and dots represent individual experiments. MOI, multiplicity of infection.

Given the observed extracellular stationary phase and envelope stress defect of the Δ*bspD* mutant, we thought to determine if this mutant’s ability to reach the intracellular replication niche and then form intracellular microcolonies was compromised. To achieve this, we infected RAW264.7 macrophages and evaluated the proportion of macrophages containing microcolonies (Fig. 4A and B; Fig. S5), as well as the size of these microcolonies after 27 h post-infection (hpi) using CellProfiler for automated image analysis (Fig. 4A and C; Fig. S5). To this end, we established a CellProfiler pipeline as previously described (34) and detailed in Materials and Methods. In brief, thresholds for identifying microcolonies were determined by comparing the size of infection sites of the replication-deficient Δ*virB9* strain with microcolonies formed by wild-type *B. abortus* in RAW264.7 macrophages after 27 h of infection (Fig. S5A and B). Using this pipeline, we observed a significant decrease in the proportion of macrophages with microcolonies upon deletion of *bspD* (Fig. 4A and B). Furthermore, the microcolonies formed were smaller compared to wild-type microcolonies (Fig. 4A and C). To determine whether the observed phenotypes (fewer infected macrophages and smaller microcolonies) resulted from reduced survival during the initial stages of infection or diminished replication in the rBCV, we infected RAW264.7 macrophages or differentiated THP-1 macrophages and lysed the host cells at 3, 27, and 48 hpi to enumerate viable bacteria (Fig. 4D through G). Differentiation of the THP-1 macrophages was confirmed on the day of infection using flow cytometry (Fig. S5C through E). At 3 hpi, the survival rates of the wild type and the T4SS-deficient mutant (Δ*virB9*) were comparable in both macrophage cell lines, whereas approximately 50% fewer bacteria were recovered from macrophages infected with the Δ*bspD* mutant (Fig. 4D through G). By 27 hpi, both the wild type and the Δ*bspD* mutant had replicated, as observed in our microscopy analysis (Fig. 4A through C). However, the CFU count for the Δ*bspD* mutant was consistently about 50% lower than that of the wild-type infected cells, a trend that persisted at 48 hpi (Fig. 4D through G). These effects were more pronounced in the THP-1 macrophages compared to the RAW264.7 macrophages. These results indicate that either not all Δ*bspD* mutant bacteria survive the early trafficking stages or fewer bacteria are internalized. Nonetheless, those that survive the initial stress are able to reach the replicative niche and replicate. These findings support our hypothesis that BspD may play a crucial role in envelope homeostasis, thereby ensuring survival in adverse environments. However, we cannot exclude the possibility that a putative effector function of BspD also contributes to the observed intracellular phenotype.

## DISCUSSION

To establish an infection as an intracellular pathogen, *Brucella* must overcome two significant challenges: firstly, it must endure various multi-stress environments, and secondly, it must manipulate the host cell to ensure its survival and replication. In this study, we present data suggesting a role for the putative T4SS effector BspD in envelope homeostasis in *Brucella*, contributing to stress resistance and survival in axenic conditions and within host cells.

BspD is preserved across Rhizobiales species with varying lifestyles, including those with and without T4SSs, except for pathogens in the *Bartonella* genus, which lack BspD, likely due to gene loss in the process of massive genome reduction in their evolutionary trajectory to blood-borne pathogens (35). This suggests that the *bspD* genes in the Rhizobiales are true orthologs derived from a common ancestor, indicating an ancient function of BspD that does not depend on the T4SS. Indeed, our observations indicate that the deletion of *bspD* results in a mild stationary phase defect. Stationary phase conditions can entail stresses such as acidification and the accumulation of reactive oxygen intermediates (18, 36). Our observations show further that the Δ*bspD* mutant experiences a notable reduction in fitness when exposed to EDTA on agar. In *Enterobacteriaceae*, EDTA disrupts the outer membrane by removing divalent cations that stabilize it via interactions with the hydrophilic part of LPS (20, 37). However, *Brucella* has a modified LPS that confers tolerance to EDTA (21, 25, 38). The fitness defect observed in the presence of EDTA, but not under osmotic stress (NaCl) or peptidoglycan stress (ampicillin), may suggest that BspD enhances the outer membrane’s tolerance to EDTA stress through modification of the non-canonical LPS. In contrast, the deletion of *eipA* affects fitness under all three stress conditions (22). Additionally, the *eipA* mutant does not replicate during infection but persists (22), whereas the *bspD* mutant can replicate, albeit at a lower level than the wild type. This indicates that while *eipA* and *bspD* may be part of the same operon, their proteins serve different, only somewhat overlapping roles in managing envelope stress.

Given that the reported lysosomal fusion to the *Brucella*-containing vacuole is limited and transient in the presence of a functional T4SS (4, 5, 8, 39) and that the fitness defect of the *bspD* mutant is mild and strongly dependent on the stressor, we hypothesize that a subpopulation of the internalized bacteria survives the acidification of the early trafficking stages. This subpopulation can then establish an intracellular niche, possibly in a delayed manner, and replicate inside the host cell once rerouted to the ER.

The *bspD* deletion mutant exhibited morphological changes when exposed to EDTA, related to those previously observed in *Escherichia coli*, including increased size and plasmolysis (40), which is in agreement with our hypothesis that BspD is involved in LPS modification in *Brucella*, leading to increased EDTA tolerance. Several genes near *bspD* contain CtrA-binding sites, such as *picC*, *ctrA*, *chpT*, *sciP*, and *mnmA* (28). These genes are crucial for *Brucella*’s growth on rich media (14) and are linked to cell cycle regulation and outer membrane composition (28, 41, 42). Furthermore, Sternon et al. reported that BspD contributes to growth on rich media but is non-essential, according to their transposon sequencing of *B. abortus* (14). This finding aligns with our results.

Besides EDTA sensitivity, the *bspD* deletion mutant showed intracellular impairments, forming fewer and smaller microcolonies than wild-type *Brucella*. This underscores BspD’s role in tolerating intracellular stress. Given BspD’s possible function as a T4SS effector, it is important to consider that its deletion might delay intracellular trafficking, leading to the observed smaller microcolonies and overall lower bacterial load. Identifying the precise reasons for these intracellular anomalies should be a priority for future studies. In this context, investigating whether a *virB9 bspD* double deletion mutant is more sensitive to intracellular degradation than the *bspD* and *virB9* single mutants might reveal a T4SS-independent role for BspD during infection. If BspD is indeed a T4SS effector, it would be the first example in *Brucella* of an effector protein with a conserved ancestral function and a newly evolved role.

## MATERIALS AND METHODS

### Bacterial strains and growth conditions

All manipulations involving live *Brucella abortus* 2308 and its derivatives were conducted in the biosafety level 3 facility at either the Swiss Tropical and Public Health Institute or the Biozentrum of the University of Basel, both located in Basel, Switzerland. These manipulations were carried out following standard operational procedures.

Bacterial strains used in this study are listed in Table 2. *E. coli* strains were cultivated in lysogeny broth or on agar plates at 37°C overnight supplemented with appropriate antibiotics.

**TABLE 2:** Strains used in this study

Strain	Relevant characteristics	Reference/source	Identifier/description
*Escherichia coli*			
Stellar	*F*−, *endA1*, *supE44*, *thi-1*, *recA1*, *relA1*, *gyrA96*, *phoA*, Φ*80d lacZ*Δ *M15*, Δ(*lacZYA-argF*) *U169*, Δ(*mrr-hsdRMS-mcrBC*), Δ*mcrA*, λ*−*	Takara Bio	Standard cloning strain
JKE201	MFDpir Δ*mcrA* Δ(*mrr-hsdRMS-mcrBC*) *aac(3)IV::lacIq*	(43)	Donor strain for conjugation
ST18	*pro*, *thi*, *hsdR*^+^, Tp^r^, Sm^r^; chromosome::RP4-2 Tc::Mu-Kan::Tn7/λ*pir*; Δ*hemA*	(44)	Donor strain for conjugation of miniTn7
JSE273	JKE201 pTNS2	This study	Helper strain for insertion of miniTn7
*Brucella abortus*			
RCBr01	2308	P. Gorvel, Centre d'Immunologie de Marseille-Luminy	
RCBr03	2308 Δ*virB9*	(45)	
MQBr19	2308 Δ*bspD*	This study	
MKBr08	2308 (*tn7*)*::P_tet_:empty*	This study	
MKBr09	2308 Δ*bspD* (*tn7*)*::P_tet_:empty*	This study	
MKBr26	2308 Δ*virB9* (*tn7*)*::P_tet_:empty*	This study	
MKBr34	2308 Δ*bspD* (*tn7*)*:: eipAbspD*	This study	
MKBr40	2308 Δ*bspD* (*tn7*)*::eipA*	This study	

*Brucella* strains were grown in TSB (Sigma-Aldrich 22092) at 37°C with 100-rpm agitation supplemented with appropriate antibiotics. Solid cultures were grown on tryptic soy agar (TSA) plates with appropriate antibiotics at 37°C.

### Cloning

All manipulations with DNA were performed following standard techniques. All cloned inserts and plasmids were sequenced to confirm integrity. For further details on plasmids and primers, see Tables 3 and 4.

**TABLE 3:** Plasmids used in this study

Plasmid	Description	Reference
pNPTS138	Cloning suicide vector encoding *sacB*, Kan^R^	Kind gift of U. Jenal (Biozentrum of the University of Basel, Switzerland)
pNPTS138_Δ*bspD* (MAR135)	Suicide vector for deletion of *bspD* from *B. abortus*	This study
pTNS2	Helper plasmid for miniTn7 genomic insertion	(46)
pUC18T-mini-Tn7T	To clone pJS203 (*tn7*)*::P_tet_:empty*	(46)
pJC44	Template for amplification of *kanR*, P*_aphT_*, *dsRed* for pJS203	(8)
pJS140	pBBR-derivative with pTetAR2, dsRed under P*_aphT_*, Cm^R^	This study
pJS203 (*tn7*)*::P_tet_:empty*	Derivative of pUC18T-mini-Tn7T, *dsRed*, Kan^R^, P*_TetAR_*	This study
pMK27 (*tn7*)*::eipAbspD*	Derivative of pJS203, expression of *eipA* and *bspD* from native promoters	This study
pMK35 (*tn7*)*::eipA*	Derivative of pMK27, expression of *eipA* from native promoters	This study

**TABLE 4:** Primers used in this study

Primer	Sequence	Purpose
prMAR59	GGATCCATGCTGGTCTATAATCTCGATG	Amplification 5′ of *bspD* for MAR135
prMAR58	GAATTCCAAAAAGAGAGCGGATTG	Amplification 5′ of *bspD* for MAR135
prMAR60	GAATTCTTTTCGCGCCAAAAGTGTG	Amplification 3′ of *bspD* for MAR135
prMAR61	GCATGCTATCCCGCTCTTGGGGAT	Amplification 3′ of *bspD* for MAR135
js428	CACCAATAACTGCCTTAAAAGCTTATATATATGAGCTCTTCACTTTTCTCTATCACTGATAGG	Amplification of *tetR*-P*_tetA_* Genescript fragment for pJS140 and pJS203
js429	ACCTATCACCTTAAATGGATATATCCCGGGATATATTTAAGACCCACTTTCACATTTAAG	Amplification of *tetR*-P*_tetA_* Genescript fragment for pJS140 and pJS203
js430	CCATTTAAGGTGATAGGTAAGATTATACC	Amplification of P*_aphT_-dsRed* fragment from pJC44 for pJS203 and pJS140
js431	CCATTTAAGGTGATAGGTATATATGGTACCATATATCTACTGGGAGCCGGAGTG	Amplification of P*aphT-dsRed* fragment from pJC44 for pJS140
js432	TTAAGGCAGTTATTGGTGCC	Linearization of pBBR1MCS for pJS140
js436	GGAGCTTGCGGCCCGGACGAATTCTCAGAAGAACTCGTCAAGAAGG	Amplification *kanR* from pJC44 for pJS203
js437	AGTAGAGCGGCCGCGATAGAGGATCGTTTCGCATGATTG	Amplification *kanR* from pJC44 for pJS203
js438	TCCTCTATCGCGGCCGCTCTACTGGGAGCCGGAGTG	Amplification of P*_aphT_-dsRed* fragment from pJC44 for pJS203
prMK025	CTTAAATGGATATATCCCGGGAACGCAGATCAGCC	Amplification of 2,160 bp *eipA* and *bspD* fragment for pMK27
prMK026	CACCAATAACTGCCTTAACTATTGCATGTCGCGGATG	Amplification of 2,160 bp *eipA* and *bspD* fragment for pMK27
prMK048	AACCCGTTCTAATAAGGCAGTTATTGGTGCCCTTAAAC	Deletion of *bspD* from pMK27 resulting in pMK35
prMK049	GCACCAATAACTGCCTTATTAGAACGGGTTCCAGGTCG	Deletion of *bspD* from pMK27 resulting in pMK35
prMK086	CCATTTAAGGTGATAGGTAAGATTATACC	Colony PCR to confirm correct insertion of miniTn7
JS436	GGAGCTTGCGGCCCGGACGAATTCTCAGAAGAACTCGTCAAGAAGG	Colony PCR to confirm correct insertion of miniTn7
prMK087	ATCATCCTCATCACCGACAAG	Colony PCR to confirm correct insertion of miniTn7
prMK088	ACCTTGAACTGCATGAACTCC	Colony PCR to confirm correct insertion of miniTn7
prMK089	GCACCAGCTTTCCGAAG	Colony PCR to confirm correct insertion of miniTn7
prMK090	CGAATTGCTGGGCACG	Colony PCR to confirm correct insertion of miniTn7

### *Brucella* in-frame deletion mutant and complementation

*bspD* was deleted in frame by double homologous recombination using the *sacB* allelic exchange suicide vector pNPTS138, which confers kanamycin resistance and sucrose sensitivity upon insertion. Excision of the insertion was counterselected on 5% sucrose TSA plates. For further details, see reference 47. The miniTn7 vector pJS203 is a derivative of pUC18T-mini-Tn7T and was used for complementation of the ∆*bspD* mutant. The *kanR* cassette was amplified from pJC44 using primers js436 and js437. The P*_aphT_-dsRed* fragment was amplified from pJC44 with primers js430 and js438. The 705-bp *tetR*-P*_tetA_* fragment was ordered as synthetic fragment from Genescript and amplified with primers js428 and js429. All three fragments were then ligated into pUC18T-mini-Tn7T digested with EcoRI and XmaI using the infusion kit (Takara). The deletion of *bspD* was complemented by cloning of a 2,160-bp long sequence encoding *bspD* and its upstream region, including *eipA* and its 5′ non-coding region harboring the CtrA-controlled promoter (22) into pJS203 after removal of the bi-directional promoter P*_tetA_* and *tetR* through digestion with SmaI and SacI. For the complementation control, *bspD* and its 5′ non-coding region were deleted from pMK27, resulting in a plasmid containing a 996-bp fragment encoding *eipA* and its 5′ non-coding region. The plasmids were finally conjugated into *Brucella* using tri-parental mating with pTNS2 as helper plasmid to ensure proper integration of the pUC18T-mini-Tn7T derivatives as described in reference 46. Proper integration into the genome at a secondary locus at the *att*Tn7 site between *glmS* and *recG* was confirmed by colony PCR and sequencing of colony PCR products with the primers listed in Table 4. Detailed description of plasmids and primers can be found in Tables 3 and 4, respectively.

### Growth curve, CFU plating, and analysis of bacterial morphology

Pre-cultures were grown overnight to mid-exponential phase (OD_600_ of 1.0–1.4) in 3 mL TSB. TSB (150 µL) with or without indicated supplements per strain in a 96-well plate (Falcon, 353072) was inoculated at OD_600_ of 0.08 (1.334 × 10^5^ CFU/mL). The plate was sealed with a gas permeable seal (Azenta Life Sciences 4ti-0516/96*) and incubated at 37°C and 237 cpm for the indicated times in a Synergy H1 plate reader (Agilent BioTek). OD_600_ was measured every 10 min. When needed, CFU per milliliter was enumerated at the endpoint. 70 microliters of culture was centrifuged at 12,000 rpm for 5 min to pellet bacteria. Bacteria were then resuspended in 140 µL phosphate-buffered saline (PBS). Seventy microliters of the resuspension was used for dilution plating. For this, the samples were serially diluted 1:5 in a 96-well plate with PBS as diluent, and 9 µL of each dilution was plated on TSA plates. Plates were incubated at 37°C for at least 3 days before counting and enumeration of CFU per milliliter. To investigate bacterial morphology, bacteria were harvested as described above. Five hundred microliters 4% para-formaldehyde (PFA) was added to each sample to fix bacteria. After 20 min of incubation at room temperature, PFA was removed by centrifugation and samples were washed once with PBS and resuspended in appropriate PBS volumes. Samples were spotted on 1.5% agarose-PBS pads, covered with a cover glass, and imaged using a Nikon Eclipse Ti2 with a Hamamatsu ORCA-Flash4.0 V3 Digital CMOS camera (C13440-20CU) using a ×100 objective [Nikon Plan Apo Lambda 100× Oil Ph3 DM (MRD31905)]. Images were analyzed using CellProfiler (version 2.2.0) (48) as described below.

### Analysis of bacterial morphology using CellProfiler

DsRed-expressing bacteria were identified as primary objects with a typical diameter of 8–50 pixel units using the “global threshold strategy” with the Otsu method and “two classes thresholding” based on dsRed. The threshold smoothing scale was set to 1.3488 with 1.0 as threshold correction factor and 0.0 and 1.0 as lower and upper bounds on the threshold. Clumped objects were distinguished by their intensity, and dividing lines were drawn accordingly. The size of the smoothing filter for declumping and the minimum allowed distance between local maxima were analyzed automatically. This led to the detection of >90% of the bacteria in any given microscopy image. The area of each object (“areashape_area”) was measured as a proxy for bacterial size based on the identified primary objects. Output files were one comma-separated value (csv) file per object category containing the selected measurements. For a visual overview, refer to Fig. S4A.

### Stress assay on plate

Stress plate assays were performed as described previously (22) with some adjustments. Bacteria were grown to stationary phase (OD_600_ of 3.0–3.6) in TSB. They were harvested by centrifugation and resuspended in sterile PBS to an OD_600_ of 0.1 (1.67 × 10^8^ CFU/mL). The resuspensions were then serially diluted as described above. Finally, 9 µL of each dilution was plated on plain TSA plates and TSA plates containing either additional 200 mM NaCl (286 mM total NaCl), 2 mM EDTA, or 5 µg/mL ampicillin. Plates were incubated at 37°C for at least 3 days before counting and calculating the CFU per milliliter.

### Mammalian cell culture

The murine macrophage cell line RAW264.7 (ATCC TIB-71TM) was grown at 37°C with 5% CO_2_ in Dulbecco’s modified Eagle medium (DMEM) + GlutaMAX (Gibco 61965–026) supplemented with 10% heat-inactivated fetal calf serum (FCS; Gibco 10270). Human THP-1 monocytes (ATCC TIB202) were cultured in RMPI1640 (Sigma Aldrich R8758) supplemented with 10% heat-inactivated FCS. Monocytes were differentiated by the addition of 20-ng/mL phorbol 12-myristate-13-acetate (PMA; Sigma Aldrich P1585) to 2 × 10^5^ monocytes/mL for 72 h, after which RPMI1640/FCS with PMA was replaced by RPMI1640/FCS. Differentiated macrophages were then incubated for another 24 h before experimentation. To ensure proper differentiation, THP-1 macrophages and monocytes were washed with PBS, lifted with trypsin if needed, collected, and fixed using 4% PFA (Boster Bio AR1068). Then the cells were washed with PBS and stained with α-CD11b antibody (Brilliant Violet 785 anti-mouse/human CD11b antibody clone M1/70, BioLegend 101243) for 30 min in PBS. Afterward, Perm/Wash Buffer (BD 554723) was added, and samples were centrifuged to remove unbound antibody. Cells were resuspended in Perm/Wash Buffer with α-CD68 antibody (Brilliant Violet 421 anti-human CD68 antibody clone Y1/82A, BioLegend 333828) for 1 h. Then cells were washed with PBS, and samples were acquired using a BD LSR Fortessa. Data were analyzed using FlowJo (BD version 10.8.1).

### Infection of RAW264.7 macrophages and imaging

One day before infection, 1 × 10^5^ macrophages/mL were seeded in 96-well plates (Corning 3904). Bacteria were grown to an OD_600_ of 1.0–1.4 (mid-exponential phase) in TSB. Bacteria were then diluted in DMEM/10% FCS to a multiplicity of infection of 50 and added to the macrophages. Plates were centrifuged at 400 × *g* for 10 min at room temperature prior to incubation at 37°C with 5% CO_2_. Two hours post infection, the infected macrophages were washed twice and further incubated in DMEM/10% FCS containing 100-µg/mL gentamicin (Sigma-Aldrich G1397) to remove extracellular bacteria. Samples were fixed 27 hpi. Before fixation, samples were washed three times with PBS to remove free bacteria. Samples were fixed with 3.7% PFA (Sigma, F1635) in 0.2 M HEPES (pH 7.2–7.4, Gibco 15630080) for 20 min at room temperature. Following incubation, samples were washed three times with PBS, and cell nuclei were stained with DAPI (1 µg/mL in PBS, Sigma-Aldrich D9542) for 15 min at room temperature. Stained samples were washed three times with PBS prior to imaging. Microscopy was performed with Molecular Devices ImageXpress automated microscopes as described in reference 34. Nuclei were imaged using DAPI. Bacteria were identified by dsRed as described below.

### CellProfiler analysis of infected RAW264.7 macrophages

Images were analyzed using CellProfiler (version 2.2.0) (48) as described in reference 34 with some adjustments. In brief, illumination was corrected using an illumination function based on the background method to adjust uneven illumination. Images were then smoothed using a Gaussian method with 100-filter size. To remove the signal originating from *Brucella* DNA in the DAPI channel, the dsRed image was subtracted from the DAPI image. Objects were segmented and measured as follows: DAPI-stained nuclei were detected as primary objects using the automatic threshold strategy. Clumped objects were distinguished using their intensity, and dividing lines were drawn according to their shape using a smoothing filter of 5. Nuclei were filtered based on their size features using a minimum measurement value of 0.1 for AreaShape with the Measurement FormFactor. DsRed-expressing bacteria were detected as microcolonies with a typical diameter of 6–30 pixels as primary objects using the “adaptive threshold strategy” followed by a “background method.” Smoothing was done “automatic” with a “threshold correction factor” of 1.35 with 0.04 lower and 1.0 upper bounds on threshold. Clumped objects were distinguished and divided using their “intensity” with a smoothing filter of 8. The cell body was estimated by expansion of the nuclei by 9 pixels. Bacteria partially or entirely within the estimated cell body were scored as 1. The number of macrophages with microcolonies and the size of the microcolonies (areashape_area) were reported for each image. To set boundaries for the identification of microcolonies, the typical diameter of *B. abortus* wild type vs Δ*virB9* mutant was determined from the measured area using dd = 2(√*A* / √π) (dd is typical diameter; *A* is area) (Fig. S5). Data from the same condition from separate wells were pooled. Output files were one csv file per object category containing the selected measurements.

### Infection of RAW264.7 and THP-1 macrophages for CFU plating

Four days before infection, 2 × 10^5^ THP-1 monocytes/mL were seeded in 96-well plates (Corning 3904) and differentiated as described above. One day before infection 0.625 × 10^5^ RAW264.7 macrophages/mL were seeded in 96-well plates. Macrophages were infected as described above. After the indicated times, the macrophages were washed with PBS and lysed with pre-warmed 0.1% Triton X-100 (in water, Sigma-Aldrich T9284) at room temperature for 10 min. Bacteria were collected by centrifugation of the cell lysate for 5 min at 12,000 × *g*. The supernatant was discarded; pellets were washed two times in PBS and finally resuspended in PBS. Recovered bacteria were serially diluted and plated on TSA plates as described above. After 3 days of incubation at 37°C, CFUs were enumerated.

### Identification of BspD orthologs, phylogenetic analysis, and synteny

The synteny for chosen species was analyzed using the SyntTax algorithm with either *bspD* (BAB1_1611) or *eipA* (BAB1_1612) of *Brucella abortus* 2308 as bait (https://archaea.i2bc.paris-saclay.fr/SyntTax/) (33). The SyntTax algorithm analyzed 15-kb DNA segments centered to the bait sequence, thereby providing information about genetic loci in various species encoded on chromosomal regions of common evolutionary ancestry (33).

The species tree of life based on the species phylogeny of the hierarchical orthologous groups (HOGs) was recovered from the OMA browser (https://omabrowser.org/oma/current/) (49) and simplified to representative species of the genus Rhizobiales. The presence or absence of *bspD* (BAB1_1611) and *eipA* (BAB1_1612) orthologs was based on their respective HOGs retrieved from OMA (49). The presence or absence of annotated VirB-T4ASSs was inferred from the SecReT4 database (https://bioinfo-mml.sjtu.edu.cn/SecReT4/index.php) (50). The different lifestyles were inferred from literature.

Sequences of BspD homologs were retrieved from the OMA browser (49) based on the OMA Group MVEPIMY of BAB1_1611 from *B. abortus* 2308. Twenty-five orthologs from different members of Rhizobiales were taken for the phylogenetic analysis presented in Fig. S1 and S2. There were no orthologs detected outside of Rhizobiales. Species and strain names are listed in Table S1. Protein sequences were aligned with Geneious Prime (version 2019.0.4) using Geneious Alignment with standard settings (Fig. S1 and Data File S1). The maximum likelihood phylogeny presented in Fig. S2 was built with Geneious using PhyML with standard settings and bootstrap 100 (51).

### Statistical analysis

Graphs were created with GraphPad Prism (version 9). Statistical analysis was performed using GraphPad Prism with one-way analysis of variance with Tukey’s multiple comparison test with *P ≤ 0.0371, **P ≤ 0.0066, ***P ≤ 0.0003, and ****P < 0.0001. When indicated, outliers were removed using the ROUT method with *Q* = 10%. The number of independent replicates is indicated in the figure legends as *n*. Growth data were fitted to extract growth dynamics with GraphPad Prism (version 9) using a Gompertz algorithm as indicated.

## Data Availability

Raw data (microscopy images and flow cytometry data) are available at 10.5281/zenodo.12779135. All numerical data can be found in Data File S2.

## SUPPLEMENTAL MATERIAL

Data File S1 msphere.00232-24-s0001.txt Multiple sequence alignment.

Data File S2 msphere.00232-24-s0002.xlsx All numerical raw data.

Supplemental material msphere.00232-24-s0003.docx Data file legends and supplemental figures and tables.

ASM does not own the copyrights to Supplemental Material that may be linked to, or accessed through, an article. The authors have granted ASM a non-exclusive, world-wide license to publish the Supplemental Material files. Please contact the corresponding author directly for reuse.
