# Supplementary material for: The putative type 4 secretion system effector BspD is involved in maintaining envelope integrity of the pathogen Brucella
Source: mSphere. 2024 Oct 10;9(11):e00232-24. doi: 10.1128/msphere.00232-24 (PMC11580434; doi:10.1128/msphere.00232-24)
Supplement: Supplemental material — Data file legends and supplemental figures and tables. [file msphere.00232-24-s0003.docx]

**Supplemental figures and tables**

**Supplementary Data File 1: Full multi-sequence alignement of protein sequences of BspD orthologues of diverse species within the Rhizobiales.** Protein sequences were aligned with Geneious Prime 2019.0.4 using Geneious Alignment with standard settings. A subset of this alignment is shown in Figure S1.

**Supplementary Data File 2: Collection of all numerical raw data including statistical tests used to generate depicted graphs and figures.**


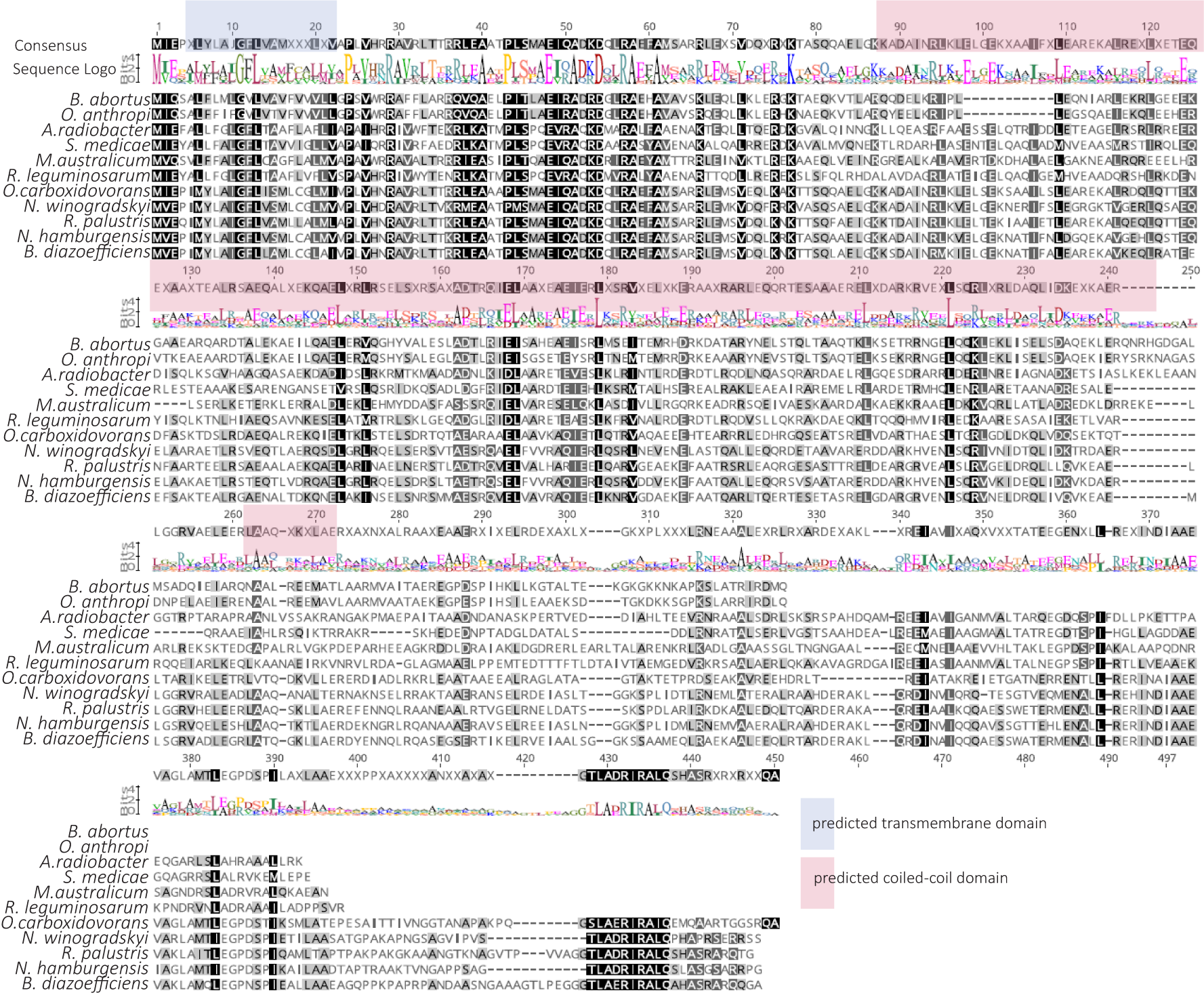


**Fig. S1: Protein sequence alignment of BspD orthologues of diverse species within the Rhizobiales.** Multi-sequence alignment of protein sequences of BspD from the following species (accession numbers indicated in brackets): *B. abortus* (BAB1_1611), *O. anthropi* (A0A6I0CU19), *A. radiobacter* (B9J8Q3), *S. medicae* (A0A508WYJ9), *M. australicum* (L0KKH9), *R. leguminosarum* (Q1MCS8), *O. carboxidovorans* (WP_012564253), *N. winogradskyi* (Q3SVA9), *R. palustris* (Q6N9C7), *N. hamburgensis* (Q1QI36), and *B. diazoefficiens* (A0A2A6MP69). Predicted transmembrane and coiled-coil domain from BspD of *B. abortus* highlighted in blue and pink, respectively. For a full aligment see supplementary data file 1.


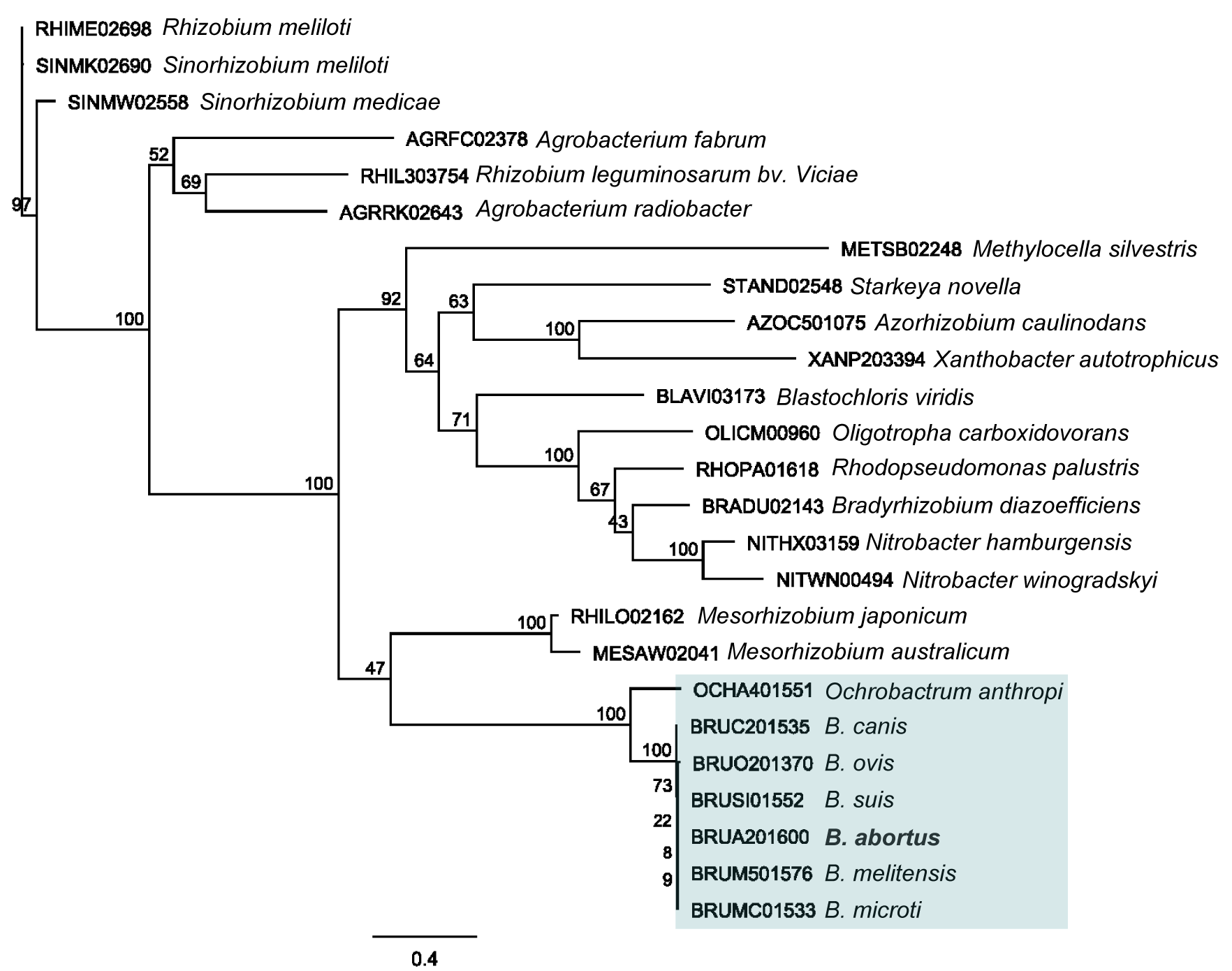


**Fig. S2: Phylogenetic tree of BspD.** Geneious PhyML tree based on alignment of 25 sequences from OMA group MVEPIMY (see table S1). Bootstrap values above 50 are shown. Line at bottom depicts 0.4 substitutions per site. *Brucellaceae* marked in teal.

**Fig. S3: CFU enumerations of stress plate assay and growth curves.** (A) Growth curve data presented in Fig. 2B fitted with the Gompertz algorithm to determine the growth dynamics of the indicated strains presented in table 1. (B) CFU enumeration after growth in TSB for 96 h. Corresponding to Fig. 2C. (C) Survival ratio in % based on counted CFU presented in (B) normalized to wild type. Corresponding to Fig 2C. **(D)** CFU enumeration after growth on TSA supplemented with indicated stressors for at least 2-3 days at 37 °C. Corresponding to Fig. 3A. **(E)** CFU enumeration at endpoint after growth in TSB supplemented with indicated concentrations of EDTA for 96 h. Corresponding to Fig 3C. Dots represent individual experiments +SD. Statistical analysis was performed with ordinary one-way ANOVA followed by Tukey’s multiple comparison test to Δ*bspD*.


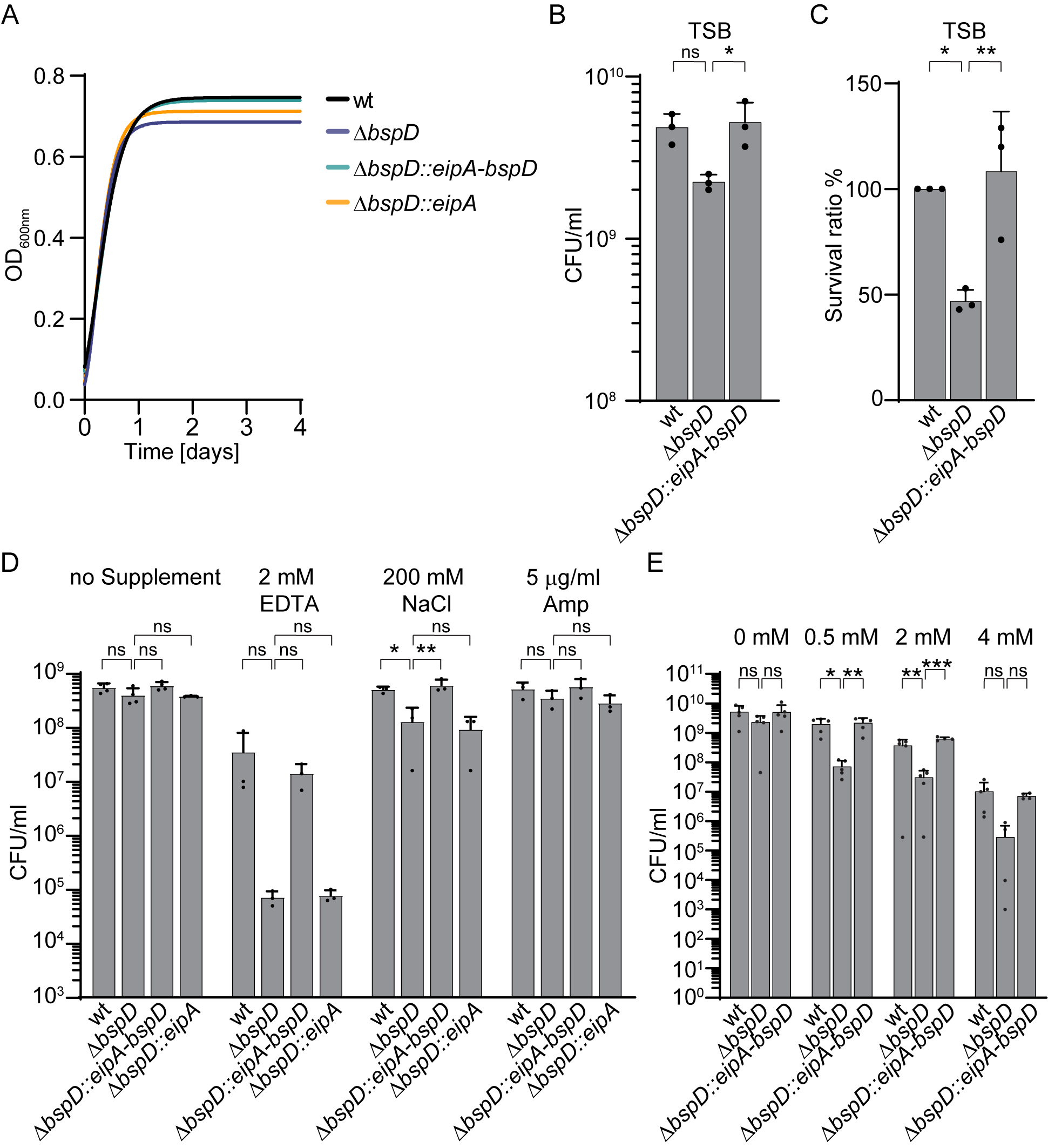

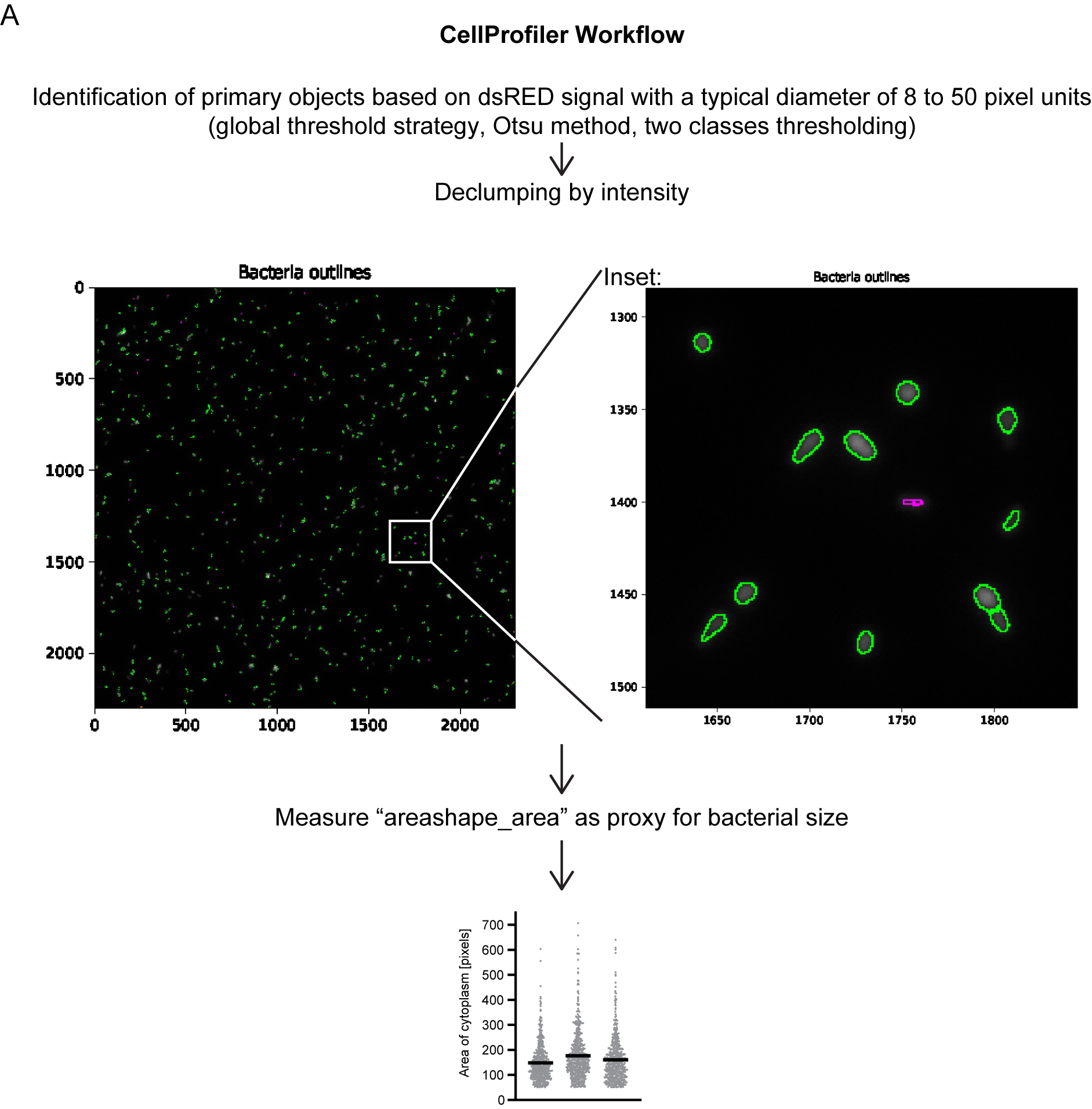


**Fig. S4: Workflow overview of CellProfiler pipeline.** After identification of primary objects based on dsRED and declumping, bacteria are masked (green) and objects that do not fit the threshold are discarded from the analysis (pink).


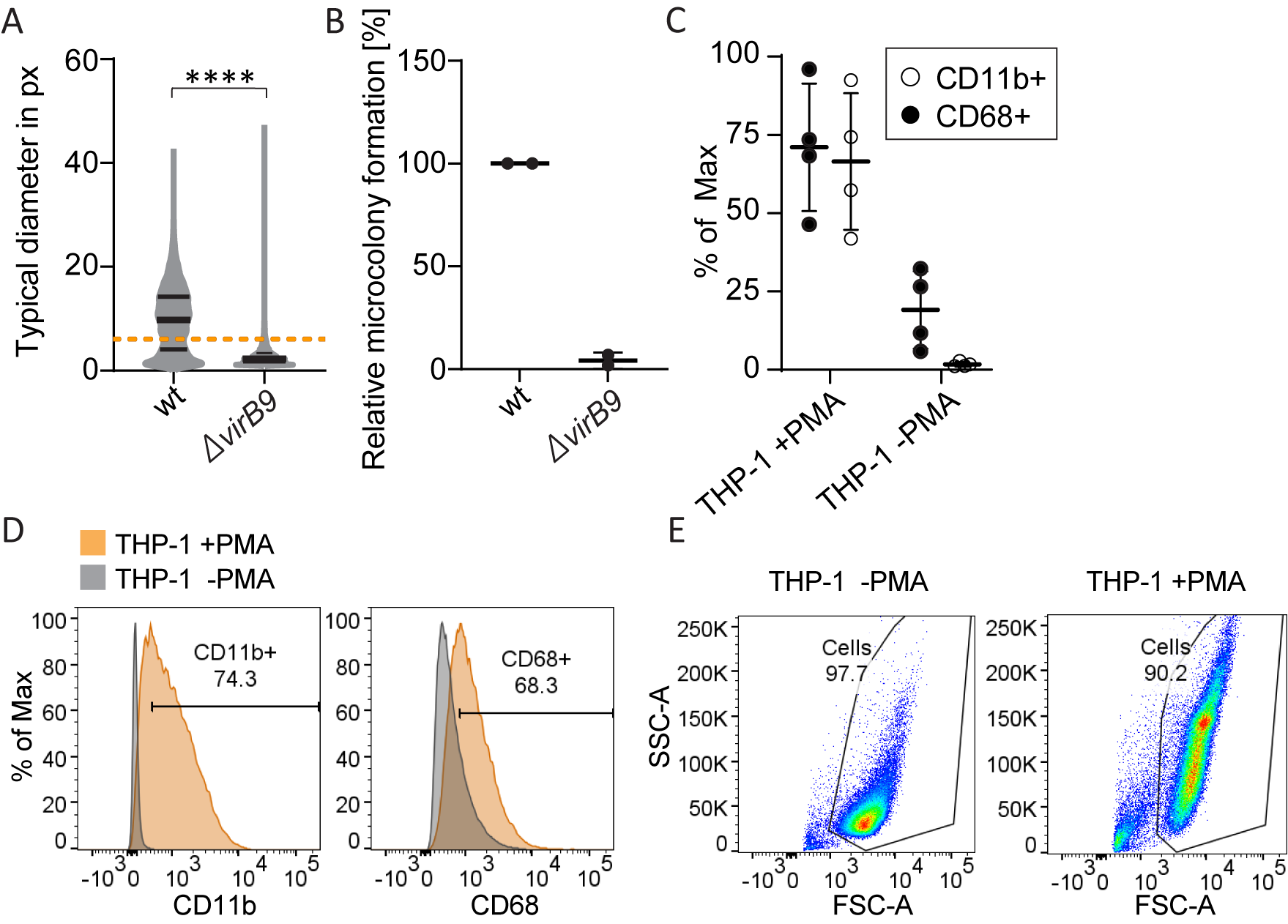


**Fig. S5: Definition of thresholds for the identification of microcolonies in infected RA264.7 macrophages (A,B) and evaluation of differentiation of THP-1 monocytes to macrophages (C-E).** **(A)** RAW264.7 macrophage-like cells were infected with an MOI of 50 for 27 hpi. After fixation and staining of host cell nuclei with DAPI, samples were imaged using a Molecular Devices ImageXpress microscope. Images were analyzed using cell profiler. The typical diameter of a circle was calculated from measured areas in pixel units (px) with the formula dd = 2(√A/√π). Orange line – 95% percentile of the ∆*virB9* population at 6.077. Black – mean. n=1 with over 17000 events analyzed. **(B)** RAW264.7 cells were infected and processed as in (A). The relative microcolony formation was measured using a CellProfiler pipeline with lower threshold for identification of microcolonies set at 6 pixels and upper threshold at 30 pixels based on the experiment presented in (A). n=2. **(C-E)** Differentiation control of THP-1 cells treated with PMA. PMA treatment of THP-1 monocytes led to a change in surface receptor expression (C, D), and a change in granularity and size of the cells (E) indicative of differentiation of monocytes to macrophages. (D) and (E) show data from one representative experiment of 4. n=4.

**Table S1: Strains used for the phylogenetic analysis of BspD orthologues of OMA group MVEPIMY**

| Accession Number | Protein ID | Species Name | Strain Identifier |
| --- | --- | --- | --- |
| A0A0H3ASC5 | BRUO201370 | *Brucella ovis* | ATCC 25840 |
| A0A0H5BNY9 | BLAVI03173 | *Blastochloris viridis* | - |
| A0A2A6MP69 | BRADU02143 | *Bradyrhizobium diazoefficiens* | JCM 10833 |
| A0A6I0CU19 | OCHA401551 | *Ochrobactrum anthropi* | ATCC 49188 |
| A0A7Y0XP19 | AGRFC02378 | *Agrobacterium fabrum* | ATCC 33970 |
| A0A222HNP4 | RHIME02698 | *Rhizobium meliloti* | 1021 |
| A0A508WYJ9 | SINMW02558 | *Sinorhizobium medicae* | WSM419 |
| A7IL20 | XANP203394 | *Xanthobacter autotrophicus* | ATCC BAA-1158 |
| A8HQZ7 | AZOC501075 | *Azorhizobium caulinodans* | ATCC 43989 |
| A9M704 | BRUC201535 | *Brucella canis* | ATCC 23365 |
| B0CI72 | BRUSI01552 | *Brucella suis* | ATCC 23445 |
| B8EIB6 | METSB02248 | *Methylocella silvestris* | DSM 15510 |
| B9J8Q3 | AGRRK02643 | *Agrobacterium radiobacter* | ATCC BAA-868 |
| BM590 | BRUM501576 | *Brucella melitensis* | M5-90 |
| C7LDI5 | BRUMC01533 | *Brucella microti* | CCM 4915 |
| D7A4M7 | STAND02548 | *Starkeya novella* | ATCC 8093 |
| L0KKH9 | MESAW02041 | *Mesorhizobium australicum* | HAMBI 3006 |
| Q1MCS8 | RHIL303754 | *Rhizobium leguminosarum bv. viciae* | 3841 |
| Q1QI36 | NITHX03159 | *Nitrobacter hamburgensis* | DSM 10229 |
| Q2YQA7 | BRUA201600 | *Brucella abortus* | 2308 |
| Q3SVA9 | NITWN00494 | *Nitrobacter winogradskyi* | ATCC 25391 |
| Q6N9C7 | RHOPA01618 | *Rhodopseudomonas palustris* | ATCC BAA-98 |
| Q98HM0 | RHILO02162 | *Mesorhizobium japonicum* | LMG 29417 |
| Sinme_2777 | SINMK02690 | *Sinorhizobium meliloti* | AK83 |
| WP_012564253 | OLICM00960 | *Oligotropha carboxidovorans* | OM4 |
